# Flooding Greatly Affects the Diversity of Arbuscular Mycorrhizal Fungi Communities in the Roots of Wetland Plants

**DOI:** 10.1371/journal.pone.0024512

**Published:** 2011-09-12

**Authors:** Yutao Wang, Yelin Huang, Qiu Qiu, Guorong Xin, Zhongyi Yang, Suhua Shi

**Affiliations:** 1 State Key Laboratory of Biocontrol, School of Life Sciences, Sun Yat-sen University, Guangzhou, Guangdong, China; 2 Key Laboratory of Ecology and Environmental Science in Guangdong Higher Education, School of Life Sciences, South China Normal University, Guangzhou, Guangdong, China; J. Craig Venter Institute, United States of America

## Abstract

The communities of arbuscular mycorrhizal fungi (AMF) colonizing the roots of three mangrove species were characterized along a tidal gradient in a mangrove swamp. A fragment, designated SSU-ITS-LSU, including part of the small subunit (SSU), the entire internal transcribed spacer (ITS) and part of the large subunit (LSU) of rDNA from samples of AMF-colonized roots was amplified, cloned and sequenced using AMF-specific primers. Similar levels of AMF diversity to those observed in terrestrial ecosystems were detected in the roots, indicating that the communities of AMF in wetland ecosystems are not necessarily low in diversity. In total, 761 Glomeromycota sequences were obtained, which grouped, according to phylogenetic analysis using the SSU-ITS-LSU fragment, into 23 phylotypes, 22 of which belonged to Glomeraceae and one to Acaulosporaceae. The results indicate that flooding plays an important role in AMF diversity, and its effects appear to depend on the degree (duration) of flooding. Both host species and tide level affected community structure of AMF, indicating the presence of habitat and host species preferences.

## Introduction

Arbuscular mycorrhizal fungi (AMF, phylum Glomeromycota) are associated with the majority of land plants [1] in a symbiosis known as arbuscular mycorrhiza (AM), which has existed for more than 400 million years [2]. In exchange for photosynthates provided by the plant symbionts, the fungal partners improve the plants' access to phosphates, nitrogen and other mineral nutrients. They also play important roles, such as improving water economy [1] and pathogen resistance [3], [4]. The composition of AMF communities can also affect the diversity and productivity of land-plant communities [5], [6]. Therefore, it is essential to research the composition and distribution of AMF in different environments.

Species- or isolate-level discrimination of active root-colonizing AMF is only possible by applying molecular methods, because the morphological features of AMF structures *in planta* do not allow for accurate identification to the species level [7]. To date, fewer than 250 morphospecies of AMF have been described (http://www.amf-phylogeny.com). Given the widespread distribution of such a relatively low number of AMF species among a large number of host species, AM fungal specificity or preference has traditionally been considered to be low. This is also supported by some studies in which low AMF specificity to host species has been observed [1], [8], [9]. However, some studies suggest that AMF are host-specific [10]–[13], and AMF have been repeatedly shown to exhibit host-specific growth responses [14] and to induce different growth responses in different host plant species [5], [15]. Overall knowledge of preferential associations of AMF with plants under natural and managed environmental conditions is still limited, and both the existence and the degree of specificity or preference of AMF remain to be resolved.

Recently, there has been increasing awareness of the occurrence of AMF in wetland ecosystems. Indeed, although the functional roles of AMF in such ecosystems are still poorly understood, it has been proposed that AMF are not only present, but ubiquitous in these habitats [16]–[18]. AMF species have also been identified from several wetland ecosystems [18], [19]. However, most of these investigations were based on the morphological characters of spores in rhizosphere soil; few focused on the composition of AMF colonizing the roots of wetland plants [20]–[24]. It has often been reported, however, that the ubiquitous presence of AMF in wetland ecosystems is closely related to the well-developed aerenchyma present in wetland plants [16], [18].

Mangrove forests are important wetland ecosystems, fulfilling essential ecological functions and harboring precious natural resources. Mangrove species grow at the interface between land and sea in tropical and sub-tropical regions with high salinity, brackish waters, and muddy, anaerobic soils, where they play very important roles in coastal ecosystem processes. They create unique ecological environments that host rich assemblages of species, and also protect and stabilize coastlines, enrich coastal waters, yield commercial forest products, and support coastal fisheries [25]. Despite the saline and microaerobic conditions in the rhizosphere of mangrove species, several studies have shown that AMF are ubiquitous in these habitats [18], [19], and there are indications that AMF could greatly improve the growth of mangrove plants through enhanced absorption of nutrient elements [18]. Since AMF need oxygen to thrive, flooding may inhibit AMF colonization, and accordingly several previous studies have found a decrease in the degree of AMF colonization with flooding along wetland gradients [16], [18]. The results of a molecular investigation indicated that flooding could even eliminate the association between AMF and the roots of a wetland species [21]. Salinity is another factor that could inhibit AMF in mangrove ecosystems. It has been reported that salinity inhibits AMF spore germination and the colonization of plant roots under laboratory conditions [26]. Wilde et al. also reported a relatively low AMF diversity within the roots of plants from two salt marshes [22]. On the other hand, spore-based studies of mangrove ecosystems, including the one investigated in this study (the Zhuhai Mangrove Area, in which only three AMF morphospecies were directly identified from 40 soil samples), have also reported comparably low AMF morphological species richness [18], [19]. However, to date, no molecular ecological investigations of AMF have been conducted in mangrove ecosystems, and the diversity and composition of AMF colonizing the roots of mangrove plants remain unknown.

The objectives of this study were to determine the AMF communities in the roots of mangrove plants and evaluate the effects of host plant species and flooding on AMF colonization and diversity in mangrove ecosystems. We measured the colonization intensity and molecular diversity of AMF associated with the roots of three mangrove species, which are all naturally distributed along a wide hydrological gradient. We hypothesized that: (i) low AMF diversity is associated with mangrove roots, and (ii) flooding by sea water greatly decreases AMF diversity in mangrove roots.

## Results

### Hydrological conditions and soil properties

The hydrological conditions and the properties of rhizosphere soil at different intertidal levels are shown in Table 1. Overall, with the exception of moisture and electrical conductivity, which are closely related to the degree of flooding and mostly showed significant increases from high tide level (HTL) to low tide level (LTL) (*P*<0.01), there were no significant differences (*P*>0.05) between the different tidal levels with respect to the soil properties, including the pH, organic matter content, and the N and P contents. Therefore it is possible to assess the effects of flooding without needing to account for soil variations at the different tidal levels.

**Table 1:** Hydrological conditions and the properties of rhizosphere soil at the high, middle and low tide levels in the Zhuhai Mangrove Area.

Tide	FH	HFL	M	EC	TP	AP	AN	pH	OM
HTL	0–0.5	1.8	31.6±1.1c	1.95±0.09b	0.41±0.01	20.7±1.3	69.3±5.0	6.84±0.28	32.4±2.1
MTL	2.0–4.0	1.0	44.8±1.1b	2.42±0.14b	0.41±0.01	19.9±1.7	69.1±5.1	7.26±0.05	32.0±3.0
LTL	7.0–10.0	0.3	62.7±1.8a	4.24±0.29a	0.44±0.01	19.0±1.4	66.3±4.2	7.11±0.12	39.0±4.2

Note: FH, estimated hours when the surface layer was under water during each 24 h period (h); HFL, estimated vertical height from the lowest tide (m); M, moisture (%); EC, electrical conductivity (ds m-1); TP, total P content (g kg-1); AP, available P content (mg kg−1); AN, available N content (mg kg−1); OM, organic matter content (%); HTL, MTL and LTL represent the high, middle and low tide level, respectively; values with different letters in the same column are significantly different at the *P*<0.05 level (mean ± SE, n = 9).

### Root colonization by AMF

All the samples examined were colonized by AMF (Table 2)**,** which formed typical AM structures. The results of two-way ANOVA (Table 3) showed that plant species had significant effects on the percent AM vesicle colonization (VC%) and arbuscular colonization (AC%) (*P*<0.001), but no significant effects on either total colonization (TC%) or hyphal colonization (HC%). The tide level significantly affected all types of AM colonization (*P*<0.001). The interactions between plant species and tide level also significantly affected the TC%, HC%, VC% and AC% (*P*<0.05). For all three investigated species (Table 2), the TC%, HC%, VC% and AC% were mostly higher at the HTL and middle tide level (MTL) than at the LTL (*P*<0.05). The AC% of *Acanthus ilicifolius* and VC% of *Acrostichum aureum* at the MTL were significantly higher than those at the HTL (*P*<0.05). In general, there were no significant differences in the TC%, HC%, VC% and AC% of different plant species from the same intertidal level (HTL, MTL and LTL**)**.

**Table 2:** AMF colonization intensity in each of three mangrove species at the high, middle and low tide levels.

Species	Tide level	TC%	HC%	VC%	AC%
*A. ilicifolius*	HTL	49.2±1.0bc	46.3±2.0bc	15.7±1.2c	17.7±1.2b
	MTL	58.7±5.8ab	50.3±5.5ab	17.7±4.2c	41.0±4.0a
	LTL	38.7±3.4c	37.0±3.6cd	10.3±3.2c	13.7±3.8bcd
*H. littoralis*	HTL	63.4±2.7a	54.5±5.5ab	38.2±4.1ab	14.7±2.6bc
	MTL	61.7±1.9a	59.0±8.2ab	40.0±2.2ab	20.3±7.2b
	LTL	23.8±3.8d	19.3±2.3e	14.9±1.4c	6.5±0.5cd
*A. aureum*	HTL	62.7±3.7a	61.7±3.2a	30.9±7.4b	11.2±3.6bcd
	MTL	68.8±4.3a	59.7±7.9ab	45.5±1.4a	14.1±0.3bc
	LTL	24.7±4.8d	23.0±5.3de	16.2±4.9c	4.5±0.3d

Note: TC%, HC%, VC% and AC% represent the percent AM total colonization, hyphal colonization, vesicle colonization and arbuscular colonization, respectively; HTL, MTL and LTL represent the high, middle and low tide level, respectively; values with different letters in the same column are significantly different across all host species at the *P*<0.05 level.

**Table 3:** Analysis of parameter estimates from generalized linear models (univariate and multivariate two-way ANOVA): effects of host, tide level and their interactions on AMF colonization intensity, phylotype richness, Shannon's index and phylotype community structure within each root sample.

Variables	TC%		HC%		VC%		AC%		N		*H*		PSC	
	F	Sig.	F	Sig.	F	Sig.	F	Sig.	F	Sig.	F	Sig.	F	Sig.
Host	0.60	ns	0.50	ns	18.17	**	15.58	**	0.56	ns	1.10	ns	10.62	*
Tide	72.68	**	37.61	**	22.74	**	21.27	**	34.31	**	21.07	**	12.00	*
Host×Tide	5.42	**	3.15	*	3.07	*	3.44	*	0.28	ns	1.26	ns	4.29	**

Note: TC%, HC%, VC% and AC% represent the percent AM total colonization, hyphal colonization, vesicle colonization and arbuscular colonization, respectively; N, phylotype richness; *H*, Shannon's index; PCS, phylotype community structure; ns, not significant at the 0.05 probability level; *, **, statistically significant at the 0.05 and 0.01 probability levels, respectively.

### PCR amplification of AMF sequences from roots

The target sequence fragments, covering part of the small subunit (SSU, *c*. 230 bp), entire internal transcribed spacer (ITS, *c*. 480 bp) and part of the large subunit (LSU, *c*. 830 bp) of the rDNA region, were successfully amplified from all root samples, and no non-specific amplification was found. Altogether, 800 clones from 27 libraries were sequenced, and a total of 761 sequences derived from Glomeromycota were obtained (39, 4.9%, potential chimeric sequences were excluded). The 761 AMF sequences were grouped into 37 operational taxonomic units (OTUs) based on sequence similarities of 97-100% by the Mothur program, and finally assigned to 23 AMF phylotypes. The numbers of AMF sequences (clones) and phylotypes detected from each mangrove species at the HTL, MTL and LTL are presented in Table S1.

### Phylogenetic analyses

**Figure 1:**
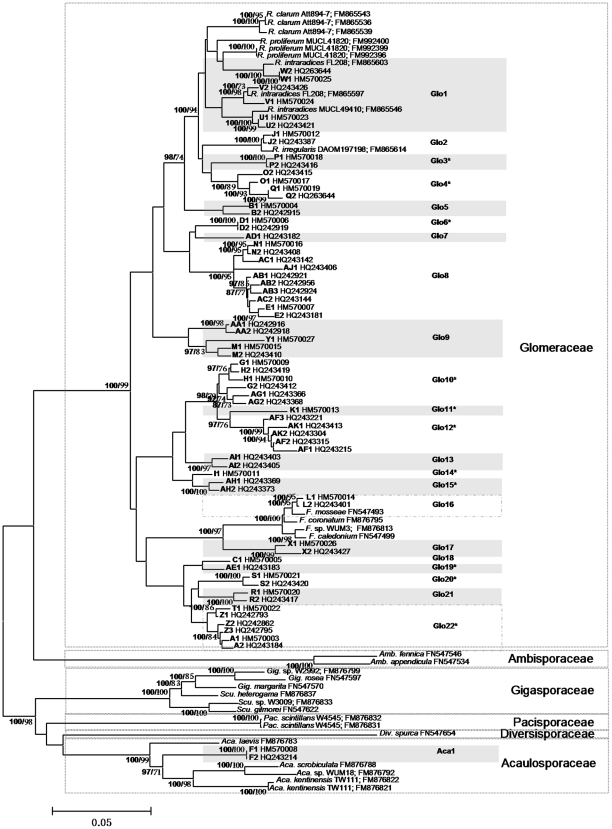
Phylogenetic relationships of the representative sequences from each OTU and the representative sequences from GenBank. The DNA fragment of AMF (partial SSU, ITS region and partial LSU rDNA sequences of approx. 1.5 kb, amplified by primers SSUmCf - LSUmBr) were obtained from roots sampled from three mangrove species in three intertidal zones, in bold. Sequences with the same alpha code (A-AK) are from the same OTUs. The asterisks are used to indicate the ‘novel’ phylotypes; the values above the branches are Bayesian posterior probabilities (bold) followed by bootstrap values (1000 replicates); only support greater than 70% is shown.

The neighbor-joining (NJ) and Bayesian analyses produced trees with the same basic topology, thus only the NJ tree is shown (Figure 1). Six families from the phylum Glomeromycota all received high support in the analyses (the applied nomenclature of Glomeromycota is from http://www.amf-phylogeny.com). The phylogenetic trees showed that among the 761 sequences (23 phylotypes) obtained in this study, 759 sequences (22 phylotypes) belonged to the family Glomeraceae, while only two sequences (one phylotype) represented the family Acaulosporaceae. This was also supported by the BLAST results. The monophyly of all Glomeraceae species detected in the present study was also highly supported by the phylogenetic analysis.

Eleven of the 23 detected AMF phylotypes, representing 367 (48%) of the 761 sequences, exhibited limited similarity (<94%, 93% and 96% in sequence similarity to the SSU-ITS-LSU, ITS and LSU fragments, respectively) to all previously published sequences. Among the other 12 AMF phylotypes (394 sequences), the AMF phylotypes Glo1 (13 sequences), Glo2 (28 sequences) and Glo16 (20 sequences) exhibited high similarity to *Rhizophagus intraradices*, *R. irregularis* and *Funneliformis mosseae*, respectively. Glo8 (282 sequences) were closely related to *Sclerocystis sinuosum* MD126 (FJ461846, partial matches in LSU). The ITS sequence of Glo17 exhibited high sequence similarity to an uncultured Glomeraceae species (98.8%, EU350770), as well as the *G. dimorphicum* BEG59 (98.4%, X96841), *F. mosseae* BEG57 (98.1%, X96834) and *G. monosporum* FM115 (98.4%, AF004690) sequences. For Aca1, the LSU region showed 97% sequence similarity to *Acaulospora foveata* CR315 (FJ461801). All the remaining phylotypes (six phylotypes, 47 sequences) were related to environmental sequences (partial matches) that were not identified to species level (Table S2).

The NJ and maximum likelihood analyses using all the sequences from Glo1-Glo4 and related sequences from GenBank produced similar phylogenetic trees, thus only the NJ tree is presented (see Figure S1). All the sequences from the same OTU are clearly clustered together in the phylogenetic tree, indicating that the grouping method based on 97% sequence similarity was appropriate.

### AMF diversity and composition in the roots

**Figure 2:**
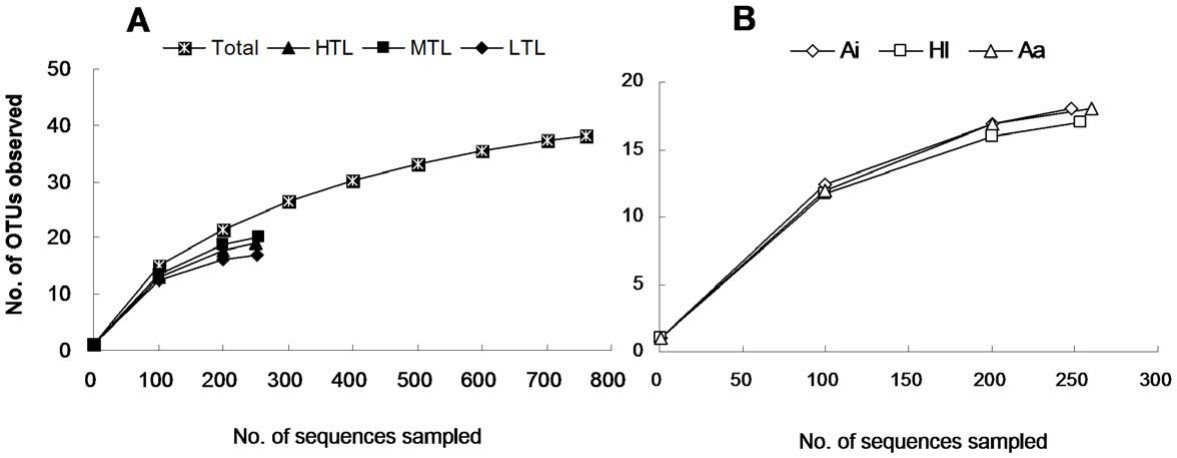
Rarefaction curves showing the sequences sampling effort of this study. (**A**) Rarefaction curves of the total number of sequences, sequences sampled from low, middle and high tide levels (LTL, MTL and HTL) and (**B**) *Acanthus ilicifolius*, *Heritiera littoralis* and *Acrostichum aureum* (Ai, Hl and Aa) at an operational taxonomic unit (OTU) threshold level of 97% sequence similarity.

As shown in Figure 2A, the rarefaction curve of the total number of obtained sequences started to level off somewhat after approximately 700 sequences, with only one more OTU included following additional sampling of 100 sequences, indicating that our sequence sampling effort detected a large proportion of the diversity of AMF. The rarefaction curves from the three tide levels (Figure 2A) and three host species (Figure 2B) also showed that we should not expect many more OTUs from further sampling. Thus, the results from the study should provide a reasonable estimate of the true AMF diversity.

**Figure 3:**
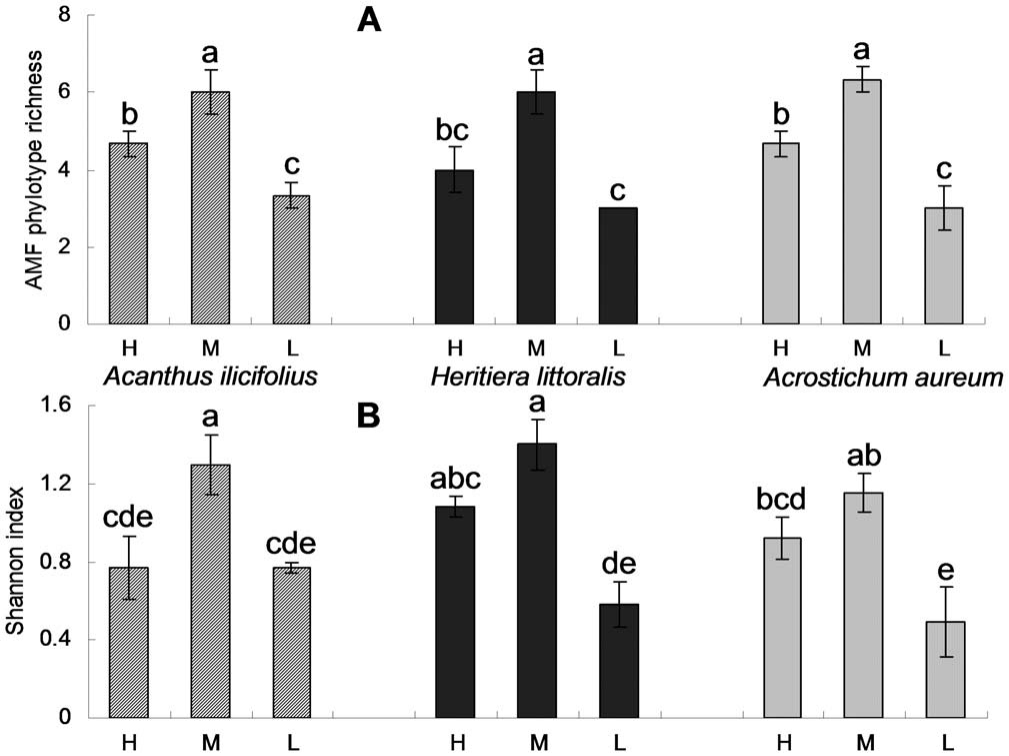
Graphical representation of the AMF (A) phylotype richness and (B) Shannon's index within roots. H, M and L represent the high, middle and low tide level, respectively. Different letters above the columns indicate significant differences across all host species and tide levels at the 0.05 level; mean ± SE.

At least two AMF phylotypes were obtained from each individual plant sample and up to seven phylotypes were found in single root samples of *Heritiera littoralis* and *Acanthus ilicifolius* from the MTL (Figure 3). Three of the AMF phylotypes (Glo8, Glo12 and Glo22), accounting for approximately 80% of the sequences obtained, were recorded in all three species from each intertidal zone, except that Glo12 was not detected in *Acanthus ilicifolius* from the HTL (Table S1). The results of two-way ANOVA (Table 3) showed that the tide level had significant effects on AMF phylotype richness and Shannon's index (*P*<0.001), while plant species and their interactions did not (*P*>0.05). In general, the phylotype richness and Shannon's index at the MTL were higher than those at the HTL and LTL (*P*<0.05), and those at the HTL were mostly higher than at the LTL (*P*<0.05) (Figure 3). The multivariate two-way ANOVA results (Table 3) showed that tide level, plant species and their interactions all significantly affected the AMF phylotype composition within the roots (*P*<0.05). They were further supported by the clustering analysis (Figure S2), in which the AMF communities from the same tide level were mostly clustered together for *Acanthus ilicifolius* and *Acrostichum aureum*; and the AMF communities from the same plant species were often closer in terms of ecological distance at all tide levels.

## Discussion

### AMF diversity in the mangrove roots

In the present study, 23 AMF phylotypes were detected in samples of 27 roots from three host species, which was unexpectedly higher than the AMF phylotype richness reportedly found in other wetland ecosystems (e.g. [22] (N<5 at two sites, based on three host species); [27] (N = 8, based on 202 roots from two host species)), and some terrestrial ecosystems (e.g. [28] (N = 11, based on 49 roots from five host species); [29] (N = 10, based on 79 root samples from four host species)). The mean numbers of AMF phylotypes detected in each root MTL and HTL sample (Figure 3A) were also higher than those detected in a semi-natural grassland (a mean of <2 phylotypes per root sample, a total of 10 phylotypes based on 80 roots from two host species, [8]) and a boreal herb-rich coniferous forest (a mean of 3.3 phylotypes per root sample, a total of 34 phylotypes based on 90 root samples from five host species, [30]). Thus, the first hypothesis in this study, i.e. low AMF diversity associated with mangrove roots, is not supported. We readily acknowledge that AMF diversity detected in different studies should be compared with caution, because many factors may affect the results of AMF diversity analyses, such as the sampling methods, criteria for defining an AMF phylotype and the molecular techniques used. For example, compared to the relatively conserved SSU and LSU fragments that have been widely used [8], [31], the SSU-ITS-LSU fragment supports higher levels of variation, which could be one of the reasons for the comparatively high diversity detected in this study. However, our results do indicate that, at least, the AMF diversities in mangrove ecosystems could be comparable to those in most terrestrial ecosystems, and that the communities of AMF in wetland ecosystems are not necessarily low in diversity. Since AMF are aerobic microorganisms, the unexpectedly high diversity we observed in the mangrove ecosystem examined could be speculatively attributed to the specialized aerial root systems and well-developed aerenchyma present in wetland plants [32], [33], including mangrove plants [34]. In addition, it is possible that some of the AMF species could have high tolerance to soil hypoxia or even anoxia. Further research is needed to identify the reasons for the apparently high AMF diversity.

The results of both the BLAST and phylogenetic analyses showed that 11 of the 23 phylotypes detected in this study were novel. Similar results have been reported in several previous studies, in which many AMF sequence types detected from natural ecosystems appeared to have no sequenced relatives among previously described AMF species [6]. There are two possible reasons why many of the phylotypes obtained in our study were novel. First, most known AMF morphospecies have not yet been sequenced, thus the rDNA sequences of some AMF species are not available in the GenBank database. Secondly, fewer than 250 AMF species have been described to date, although molecular evidence now indicates that overall AMF diversity has been severely underestimated [35]–[38]. The relatively high AMF diversity observed in this study also imply that in time many more AMF species will be discovered.

### Effects of flooding on AMF diversity and possible mechanisms

Although several previous studies have examined the effects of habitat type [31], [39], management intensity [30], [40] and soil fertilization [8] on the diversity of root-colonizing AMF, there has been no previous investigation of the influence of flooding on AMF diversity in roots. Our study showed that flooding has a highly significant effect (*P*<0.001) on AMF diversity (based on phylotype richness, Shannon's index and phylotype composition), and the effects are dependent on the degree of flooding. Thus, the second hypothesis of this study, i.e. that flooding by sea water greatly decreases AMF diversity, is only partly supported. Similar patterns of flooding effects were observed for AMF colonization intensity, implying that flooding affects both the colonization intensity and diversity of AMF by similar mechanisms.

Intensive flooding (hydrological conditions at the LTL, Table 1) was clearly associated with decreased diversity and colonization of the roots of all three mangrove species. Since AMF require aerobic conditions to thrive, this may be because they can survive in the oxygenated portions of mangrove plant roots, but are inhibited by the scarcity of oxygen outside the rhizosphere in frequently flooded zones of the mangrove ecosystem. Thus, AMF may fail to germinate from spores or colonize new roots from existing points of infection, or may do so more slowly in such zones, resulting in a lower colonization intensity and diversity of AMF in roots. Such inhibition caused by flooding is likely to be further strengthened by the high salinity of the sea water, which also inhibits AMF colonization [26].

No inhibitory effects of moderate flooding (hydrological conditions at the MTL, Table 1) on the diversity and colonization intensity of AMF were observed in any of the three plant species investigated. On the contrary, the AMF diversity level in all three species was even higher at the MTL than that at the HTL (*P*<0.05). There are three possible explanations for this result. First, the AMF colonizing the roots at the MTL might have a relatively high tolerance to salinity, as discussed above. Secondly, it has been shown that moderate flooding (e.g. 2–4 hours per day) can increase the number of pneumatophore roots and improve the efficiency of aerenchyma in mangrove plants [41]. This could largely compensate for the decreased oxygen concentrations in the rhizosphere resulting from moderate flooding. Thirdly, moderate flooding could also promote the growth of mangrove plants by enhancing their photosynthetic rates [41]. Thus, under a moderate flooding regime, mangrove plants could provide more carbohydrates and hence support a higher colonization intensity and diversity of AMF communities in their roots. Further research is needed to identify the mechanisms involved.

### AMF species composition in mangrove roots

The majority of sequences detected in the roots belonged to the family Glomeraceae. This finding is in accordance with results of a previous study based on spore morphology [18]. Many other studies on the molecular diversity of AMF have also found Glomeraceae to be the predominant family in the roots of various plants [38], [42], including wetland plants [21], [22]. Although the reasons for the predominance of this family in the mangrove ecosystem are unknown, it seems reasonable to believe that Glomeraceae could be a widespread family in wetland ecosystems, as it is in terrestrial ecosystems.

Two of the three AMF species, *R. intraradices* and *F. mosseae*, which were identified from the spores collected at the same site [18], were also detected within the roots of mangrove plants. According to Stockinger et al. [43], the *R. intraradices* sequences obtained in this study are the first true record of *R. intraradices* outside Florida, as the widespread ‘*R. intraradices*’ which has been detected in diverse ecosystems on several continents, is not *R. intraradices* but belongs to the *R. irregularis*.

Three phylotypes (Glo8, Glo12 and Glo22) were found to be dominant in all three mangrove species at all intertidal levels. We acknowledge that the relative abundance of clones should be used with caution as a proxy for the relative abundance of AMF associates, as PCR and cloning biases can influence apparent clone abundances [44], [45]. One concern in this study was the potential for primer bias of a single sequence group causing false dominance of it in those samples. However, a primer or cloning bias would show up in every sample in which that group was present. The three most abundant phylotypes, Glo8, Glo12 and Glo22, showed no such bias. Thus, clone-relative abundance should provide reasonable estimates of the relative abundance of the dominate phylotypes.

The host species had no significant effects on the diversity of AMF within roots of all three mangrove species (*P*>0.05). This is in accordance with results reported from a coniferous forest, in which the effects of host species identity on the number of AMF phylotype per plant individual was not significant (*P*>0.05) [30]. So far, differences in the number of AMF phylotype associated with host plant species have not been demonstrated [30]. On the other aspect, the results from both the two-way ANOVA and the clustering analysis clearly showed that the host species greatly affected the AMF composition within roots (*P*<0.05), indicating the presence of host species preference. This is consistent with several previous reports, in which host species influenced AMF composition in roots [11], [38]. Schechter and Bruns [31] concluded that host preference could have a strong influence on AMF composition. On the other hand, no obvious AMF host plant specificity was detected with respect to the three mangrove species as three phylotypes were dominant in all the mangrove species investigated. Some rare phylotypes appeared in just one of the host species, but they occurred at such low frequencies that no conclusions can be drawn from this dataset regarding their possible host specificity. Although advances have been made in identifying both host preference and specificity in certain natural ecosystems [10], [37], there are still many difficulties in using molecular techniques to study the specific and preferential interactions between AMF and their plant hosts [46]. It is possible that AMF host species specificity and preference are dependent on the particular species of AMF, host plant and habitat type involved. However, since little is currently known about the AMF composition in the roots of mangrove species or other wetland plants, further research is needed to identify the true implications of these results.

### Molecular approach

In this study, a set of PCR primers (SSUmAf/SSUmCf - LSUmAr/LSUmBr, [47]) was used in a field-based investigation of AMF communities associated with roots. Recently, the target fragment (SSU-ITS-LSU) of these primers has been recommended as a DNA barcoding region for AMF [47], [48]. Our results show that all of the 800 sequences we sequenced, including the 39 potential chimeric sequences, were of AMF origin, indicating the high specificity of the primers. In a preliminary experiment preceding this study, we used the widely applied primer pair NS31-AM1 [28] and a recently published primer pair, AML1-AML2 [49], to amplify AMF sequences from roots. However, most of the sequences amplified by both of these primer pairs belonged to certain unknown marine fungi (data not shown). On the other hand, the results of phylogenetic analysis using the SSU-ITS-LSU fragment showed that the topology of the phylogenetic trees obtained is generally consistent with those previously published [11], [39], [48], [50], and five families from the phylum Glomeromycota were all strongly supported. The results of the additional phylogenetic analysis also supported the viewpoint that the SSU-ITS-LSU fragment is helpful for the molecular characterization of AMF, as it contains high levels of variation, which might allow resolution at species level [47], [48].

## Materials and Methods

### Ethics Statement

All observational and field work was approved by the Qi Ao Mangrove Nature Reserve committee and performed conforming to the Regulations of the People's Republic of China on Nature Reserves.

### Study site and sample collection

The study site, the Zhuhai Mangrove Area (22°23′–22°27′ N, 113°36′–113°39′ E), is situated in the Qi Ao Mangrove Nature Reserve, Guangdong province, China (P.R.), on the estuary of the Pearl River, which is the largest and most important river in South China. Geographically, it is located close to the northern limit of mangrove distribution, in an estuarine system with an irregular semi-diurnal tide, i.e. two irregular high tides per 24 h period, with a mean tidal range of 1.9 m. The average annual precipitation, daily temperature and water salinity are 1964 mm, 22.4°C and 15‰, respectively [18]. Three dominant mangrove species – *Heritiera littoralis*, *Acrostichum aureum* and *Acanthus ilicifolius* – are naturally distributed along wide hydrological gradients here, allowing the assessment of both flooding and plant species effects on the colonization intensity and molecular diversity of AMF.

For each investigated species, root samples and rhizosphere soil samples of three individual plants in each of three intertidal zones (low, middle and high tidal level, see Table 1) were separately collected in December 2009, yielding nine root samples and nine soil samples per plant species and a total of 27 root samples and 27 soil samples. Juvenile nutritive roots attached to the plants were collected as the root samples. The sampled replicates were separated by a distance of more than 10 m.

### Soil analysis and assessment of AM colonization

The soil samples were passed through a 0.25-mm sieve before determining the organic matter content and total N; samples used to determine pH, electrical conductivity, available N and P were passed through a 1-mm sieve. The pH was measured in a 1∶2.5 soil:water paste (w/v), using a digital pH meter (Basic PB-20, Sartorius AG, Goettingen, Germany). Electrical conductivity was measured in the centrifuged supernatant of a 1∶5 soil:water (w/v) extract. Organic matter content was determined by the Walkley-Black acid digestion method. Available N (extracted by 2 M KCl) was measured by a titration of the distillates after Kjeldahl sample preparation and analysis. Total P (digested with HNO3) and available P (extracted by 0.05 M HCl - 0.025 M H2SO4) were measured by molybdenum blue colorimetry. These analyses were all based on the methods described by Page et al. [51].

Fine root samples were cleared in 10% w/v KOH at 90°C for approximately 40 min, and then stained with trypan blue. Percentage root colonization was quantified using the magnified intersection method [52] and we scored 200 intersects on 40 root segments per root sample using a compound microscope (Carl Zeiss, Axiostar plus, Germany) at 100× magnification.

### DNA extraction and PCR

Twenty root fragments (1–2 cm in length) from each root sample were chosen randomly, and mixed together for DNA extraction. Genomic DNA was extracted from the pooled root fragments using cetyltrimethylammonium bromide (CTAB) [53]. DNA extracts were then used for PCR after 1∶10 or 1∶100 dilution with distilled water.

A nested PCR was applied to amplify a fragment, designated SSU-ITS-LSU, covering part of the SSU, the whole ITS and part of the LSU rDNA region; four primer mixtures (SSUmAf, SSUmCf, LSUmAr and LSUmBr) were used, each targeting one binding site in the SSU or LSU rDNA [47]. SSUmAf-LSUmAr primers were used for the first round of PCR, which was performed with 20 µl reaction mixtures, containing 2 µl of DNA template, 2 µl of 10× PCR buffer, 1.5 µM of MgCl2, 200 µM of each dNTP, 0.5 µM of each primer and 0.05 U µl-1 TaKaRa LA Taq™ DNA Polymerase which has proof-reading activity (Takara, Japan). The amplification program was as follows: 4 min initial denaturation at 94°C; 40 cycles of 30 s denaturation at 94°C, 30 s annealing at 60°C and 100 s elongation at 72°C; followed by a 10 min final elongation. The first PCR products were diluted 1:10 and used as the template for a second (nested) round of PCR, involving 50 µl reaction mixtures, and SSUmCf-LSUmBr primer pairs. The same conditions were applied as in the first-round PCR, except that the reaction mixtures were subjected to only 32 cycles in the nested PCR. Portions (1.5 µl) of the PCR products were analyzed by agarose gel electrophoresis (1.0% w/v agarose, 100 V, 40 min), and ethidium bromide staining to check integrity and yield.

### Cloning and sequencing

The second-round PCR products with the expected length for AMF (approx. 1500 bp) were first purified using a High Pure Kit (Pearl, China) following the manufacturer's protocol. They were then cloned into the pMD-18T vector (Takara, Japan) according to the manufacturer's instructions and heat-transformed into high efficiency competent cells of *Escherichia coli* (strain DH5α, Takara, Japan). PCR amplification using the vector-specific primers M13F-M13R was applied in order to screen for putative positive transformants (PCR conditions: 25 cycles at 94°C for 30 s, 55°C for 30 s, 72°C for 90 s). Approximately 30 (N≥28) positive clones were randomly selected from each sample to construct a SSU-ITS-LSU library. All clones from each library were then directly sequenced in both directions using M13F and M13R primers. Sequencing reactions were carried out using an ABI PRISM 3730XL automatic sequencer with a BigDye Terminator V3.1 Cycle Sequencing Kit (Applied Biosystems, USA) according to the manufacturers' instructions.

### Sequence analyses and construction of phylogenetic trees

The forward and reverse sequences from each clone were first assembled into a consensus sequence, and then all the consensus sequences were proofread and trimmed with Lasergene SeqMan (DNAStar Inc.) to remove the vector sequence. The sequences obtained were compared with those available in the GenBank database using the BLAST tool to determine whether they were derived from Glomeromycota (based on the origin of the best-scoring hit in the GenBank). They were then screened for possible chimerical origin using the program Mallard, following the instructions provided on the Mallard website (http://www.bioinformatics-toolkit.org/Mallard/index.html) [54], and the BLAST tool. Because of the high genetic diversity among AMF spores of the same species, and even from single spores [55], and the relatively large sequence dataset in this study, before applying phylogenetic analysis, all AMF sequences were grouped into OTUs with sequence similarities ≥97%, using the Mothur program [56]. According to intra- and inter-specific variation data presented by Stockinger et al. [48], this should not result in single OTUs representing several species. A similar grouping method has also been reported in several previous studies [31], [38]. Rarefaction curves computed by the Mothur program, employing a permutation-based method that uses sampling without replacement, were applied to determine if the clone sampling effort had encompassed most of the OTUs. Two or three representative sequences were separately selected from each non-dominant and dominant OTU for phylogenetic analysis. ClustalX 1.83 [57] was used to examine multiple alignments of representative sequences from each OTU and all the representative Glomeromycota sequences obtained from GenBank. The sequence alignment is presented in the Alignment S1.

Two separate phylogenetic analyses were performed: NJ was conducted using MEGA 4 [58], and Bayesian analysis was performed using MrBayes 3.7 [59]. Molecular evolutionary models for Bayesian analysis were estimated with ModelTest [60]. The best-fit model was GTR + I + G (–Ln L = 26 839). Bayesian analysis was performed with four Markov chain Monte Carlo simulations over 40 million generations with trees sampled every 1000 generations for two runs. A 50% consensus tree was constructed after excluding the first 25% of trees (burn-in), and posterior probabilities were estimated for the remaining sampled generations. The reliability of clades in the NJ analysis was assessed using nonparametric bootstrapping in MEGA (Kimura's two-parameter, K2P, model; 1000 replicates).

The whole SSU-ITS-LSU fragment, the ITS region and the LSU region of the representative sequences from each OTU were separately compared with those available in GenBank using the BLAST tool to identify sequences with close matches to identified AMF species and environmental sequences (≥94% sequence similarity for the SSU-ITS-LSU fragment, ≥70% query coverage and ≥93% sequence similarity for the ITS region, ≥70% query coverage and ≥96% sequence similarity for the LSU region). We defined ‘phylotypes’ in this study mainly according to the topology of the phylogenetic tree, but the BLAST results and the average pairwise distance (calculated based on the K2P model using MEGA 4) between different OTUs were also considered when it was difficult to decide whether two phylogenetically adjacent sequences or monoclades should be placed in the same phylotype or not. Two sequences or monoclades were separated into different phylotypes when they were related to different identified AMF species and their pairwise distance was greater than 0.055. All the sequences obtained from this study were deposited in GenBank under accession numbers HM570003-HM570027, HQ242793-HQ243427 and HQ263643-263644.

To investigate further the relationships between several controversial phylotypes and their related species, an additional phylogenetic analysis was performed with RAXML [61] and MEGA [58], using all the sequences from the objective phylotypes and their well defined relative sequences. The sequences were aligned automatically using MAFFT software [62]. The sequence alignment is presented in the Alignment S2. A NJ-tree was constructed as mentioned above. RAXML was set to research maximum likelihood for the best-scoring tree after 1000 bootstraps; the proportion of invariable sites was also estimated by the program.

### Statistical analysis

AMF phylotype richness (N) was calculated as the number of phylotypes recorded in each sample. The Shannon's index (*H*) [63] of AMF communities was calculated for each sample using the equation 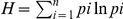


where *pi* is the relative abundance of the i-th phylotype among all phylotypes in the respective sample. A parametric one-way ANOVA, followed by a least significance difference (LSD) test at the 0.05 confidence level, was used to determine differences in the soil properties among the different intertidal levels, and the AMF colonization intensity, phylotype richness and Shannon's index among the different host plants and intertidal zones. Two-way ANOVA was applied to analyze the effects of plant species and flooding (intertidal zones) on colonization intensity, phylotype richness and Shannon's index. Multivariate two-way ANOVA, using generalized linear models, was also applied to analyze the effects of flooding and host species on the AMF phylotype compositions within roots. To decrease the influence of the dominant phylotypes, the phylotype matrix was first transformed using a double square-root function. These statistical analyses were conducted in SPSS 16.0. A clustering method was also applied to examine, in more detail, the effects of host species and tide level on the AMF phylotype compositions within roots. The hierarchical clustering analysis (agglomerative clustering algorithms) based on the Bray-Curtis ecological distances between AMF communities was used to test the similarity of AMF phylotype structures, using the “vegan” package from the R program [64].

## Supporting Information

Figure S1 Phylogenetic tree derived from neighbor-joining (NJ) and RAxML analyses based on a MAFFT alignment, showing the phylogenetic relationships of all obtained sequences from phylotypes Glo1–Glo4 and their related sequences in GenBank. AMF sequences (partial SSU, ITS region and partial LSU rDNA sequences of approx. 1.5 kb, amplified by primers SSUmCf - LSUmBr) labeled with the same symbols are from the same OTU based on a 97% sequence similarity threshold; the values above the branches are bootstrap values (1000 replicates) from maximum likelihood (in bold) and NJ analyses, respectively; only support greater than 70% in both analyses is shown.
(TIF)

Figure S2 Dendrogram of hierarchical clustering analysis based on the Bray-Curtis ecological distances among AMF communities within the roots (A) from the same host species and (B) from the same tide level. HTL, MTL and LTL represent high, middle and low tide level, respectively; Ai, Hl and Aa represent *Acanthus ilicifolius*, *Heritiera littoralis* and *Acrostichum aureum*, respectively.
(TIF)

Table S1 Abundance matrix of AMF sequences (clones) observed in each AMF phylotype within the roots of *Acanthus ilicifolius*, *H. littoralis* and *Acrostichum aureum* at the high, middle and low tide levels.
(DOC)

Table S2 BLAST results for the unidentified environmental sequences from GenBank related to the phylotypes obtained in this study.
(DOC)

Alignment S1 Sequence alignment of the representative sequences from each OTU obtained in this study and the representative AMF sequences from GenBank.
(TXT)

Alignment S2 Sequence alignment of the sequences from phylotypes Glo1–Glo4 and their related sequences in GenBank.
(TXT)
