# Supplementary figures and images for: Flooding Greatly Affects the Diversity of Arbuscular Mycorrhizal Fungi Communities in the Roots of Wetland Plants
Source: PLoS One. 2011 Sep 12;6(9):e24512. doi: 10.1371/journal.pone.0024512 (PMC3171463; doi:10.1371/journal.pone.0024512)

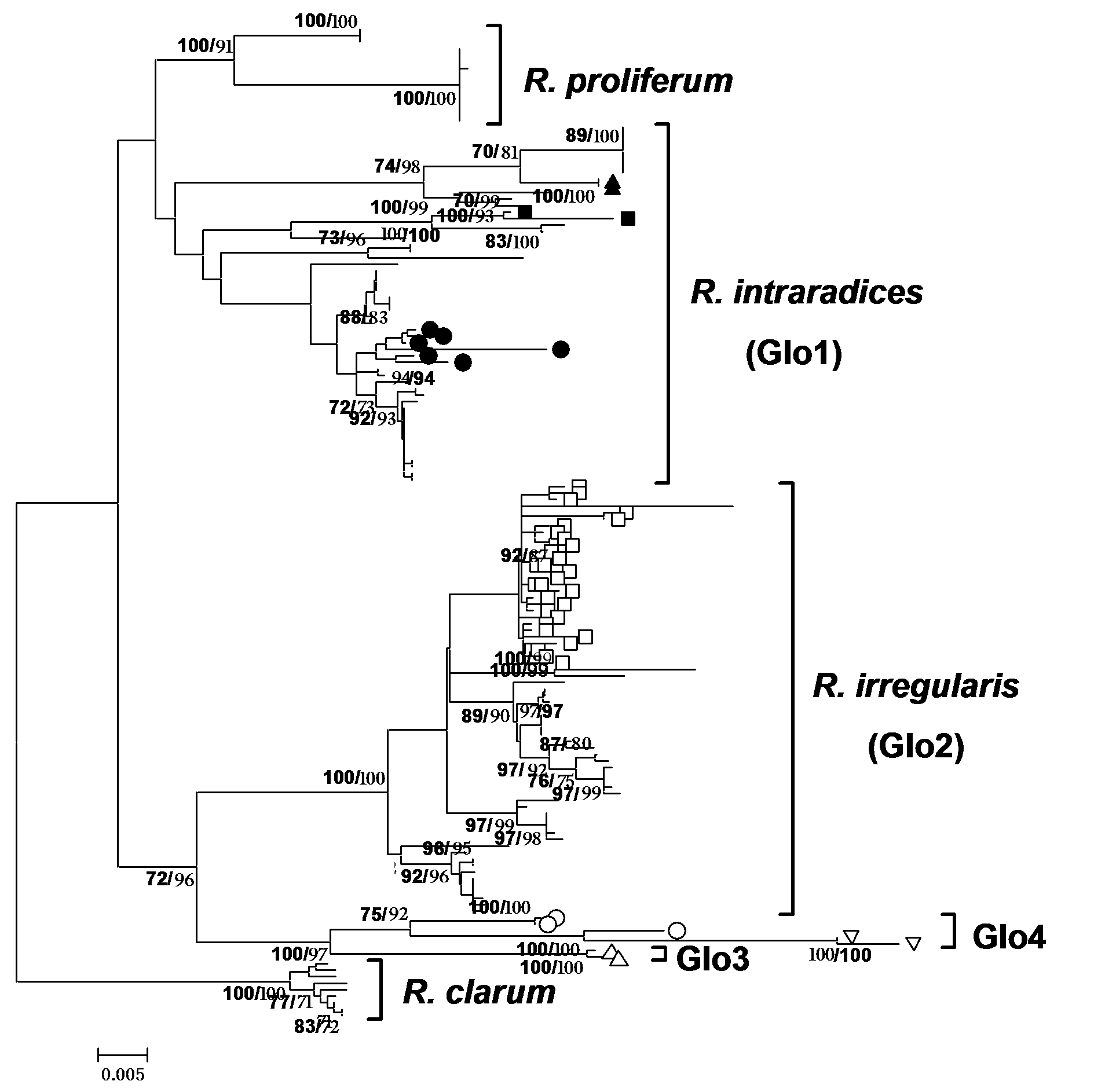

Supplement: Figure S1 — Phylogenetic tree derived from neighbor-joining (NJ) and RAxML analyses based on a MAFFT alignment, showing the phylogenetic relationships of all obtained sequences from phylotypes Glo1–Glo4 and their related sequences in GenBank. AMF sequences (partial SSU, ITS region and partial LSU rDNA sequences of approx. 1.5 kb, amplified by primers SSUmCf - LSUmBr) labeled with the same symbols are from the same OTU based on a 97% sequence similarity threshold; the values above the branches are bootstrap values (1000 replicates) from maximum likelihood (in bold) and NJ analyses, respectively; only support greater than 70% in both analyses is shown. (TIF) [file pone.0024512.s001.tif]

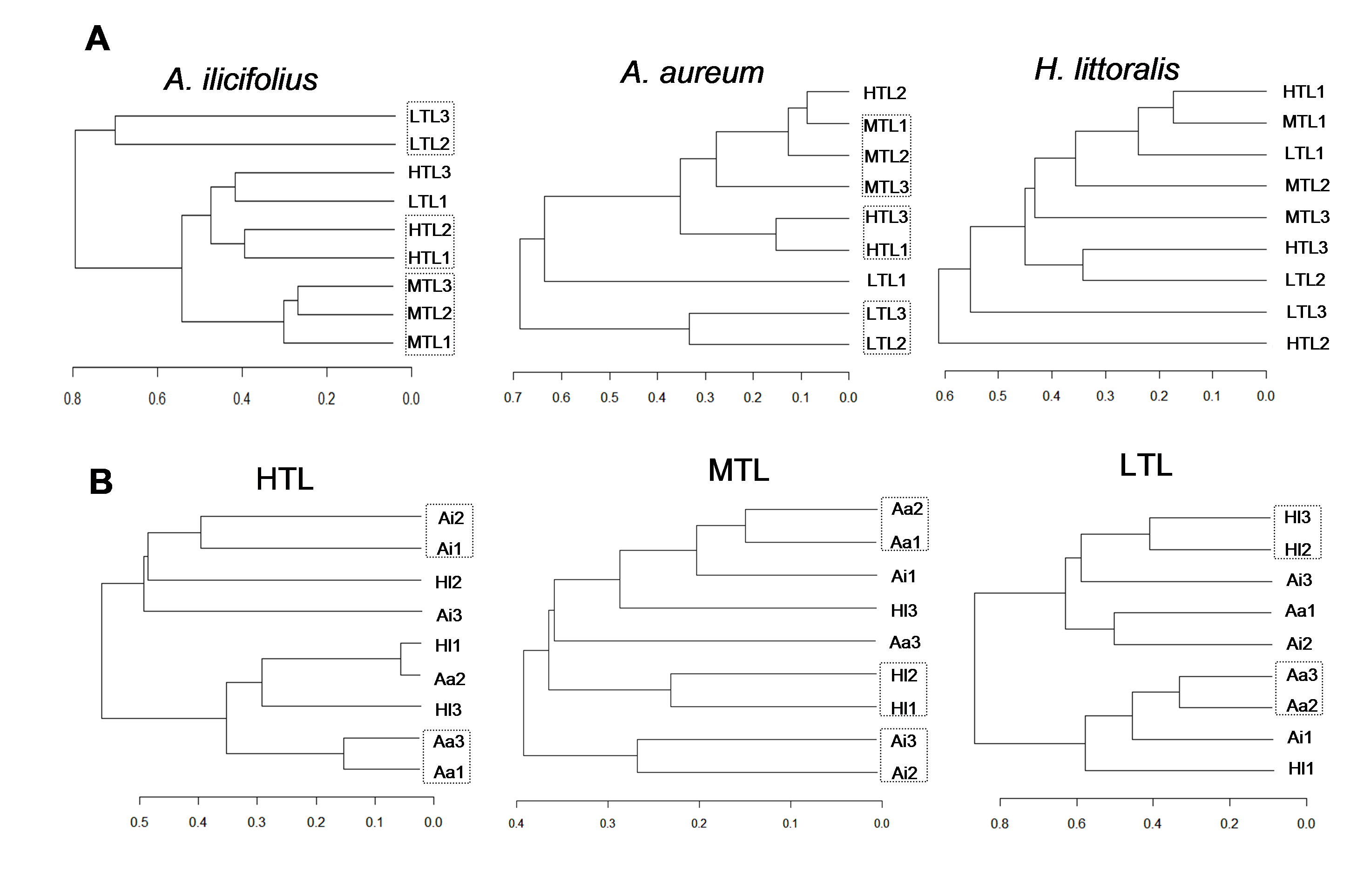

Supplement: Figure S2 — Dendrogram of hierarchical clustering analysis based on the Bray-Curtis ecological distances among AMF communities within the roots (A) from the same host species and (B) from the same tide level. HTL, MTL and LTL represent high, middle and low tide level, respectively; Ai, Hl and Aa represent Acanthus ilicifolius, Heritiera littoralis and Acrostichum aureum, respectively. (TIF) [file pone.0024512.s002.tif]
