# Supplementary material for: Flooding Greatly Affects the Diversity of Arbuscular Mycorrhizal Fungi Communities in the Roots of Wetland Plants
Source: PLoS One. 2011 Sep 12;6(9):e24512. doi: 10.1371/journal.pone.0024512 (PMC3171463; doi:10.1371/journal.pone.0024512)
Supplement: Table S1 — Abundance matrix of AMF sequences (clones) observed in each AMF phylotype within the roots of Acanthus ilicifolius, H. littoralis and Acrostichum aureum at the high, middle and low tide levels. (DOC) [file pone.0024512.s003.doc]

**Table S1** Abundance matrix of AMF sequences (clones) observed in each AMF phylotype within the roots of *Acanthus ilicifolius*, *H. littoralis* and *Acrostichum aureum* at the high, middle and low tide levels

|  | Tide |  |  |  |  |  |  |  |  |  |  |  |
| --- | --- | --- | --- | --- | --- | --- | --- | --- | --- | --- | --- | --- |
|  | HTL |  |  |  | MTL |  |  |  | LTL |  |  | **Total** |
| Species | Ai | Hl | Aa |  | Ai | Hl | Aa |  | Ai | Hl | Aa |
| Glo1 | 0 | 13 | 0 |  | 0 | 0 | 0 |  | 0 | 0 | 0 | 13 |
| Glo2 | 0 | 0 | 0 |  | 0 | 0 | 0 |  | 28 | 0 | 0 | 28 |
| Glo3 | 0 | 1 | 0 |  | 0 | 0 | 0 |  | 0 | 0 | 1 | 2 |
| Glo4 | 0 | 0 | 0 |  | 0 | 0 | 0 |  | 0 | 0 | 8 | 8 |
| Glo5 | 1 | 0 | 2 |  | 0 | 2 | 0 |  | 0 | 0 | 0 | 5 |
| Glo6 | 2 | 0 | 0 |  | 0 | 0 | 0 |  | 0 | 0 | 0 | 2 |
| Glo7 | 1 | 0 | 0 |  | 0 | 0 | 0 |  | 0 | 0 | 0 | 1 |
| Glo8 | 12 | 48 | 47 |  | 28 | 50 | 43 |  | 2 | 43 | 7 | 280 |
| Glo9 | 1 | 0 | 1 |  | 1 | 2 | 1 |  | 0 | 1 | 0 | 7 |
| Glo10 | 0 | 2 | 1 |  | 1 | 1 | 3 |  | 1 | 0 | 1 | 10 |
| Glo11 | 0 | 0 | 0 |  | 0 | 0 | 0 |  | 1 | 0 | 0 | 1 |
| Glo12 | 0 | 6 | 22 |  | 16 | 18 | 29 |  | 25 | 36 | 25 | 177 |
| Glo13 | 0 | 0 | 0 |  | 3 | 0 | 0 |  | 0 | 0 | 0 | 3 |
| Glo14 | 0 | 0 | 0 |  | 0 | 0 | 1 |  | 0 | 0 | 0 | 1 |
| Glo15 | 0 | 0 | 0 |  | 6 | 1 | 0 |  | 1 | 0 | 0 | 8 |
| Glo16 | 2 | 1 | 1 |  | 3 | 2 | 3 |  | 7 | 1 | 0 | 20 |
| Glo17 | 0 | 0 | 0 |  | 0 | 0 | 0 |  | 0 | 2 | 0 | 2 |
| Glo18 | 0 | 0 | 0 |  | 0 | 1 | 0 |  | 0 | 0 | 0 | 1 |
| Glo19 | 1 | 0 | 0 |  | 0 | 1 | 0 |  | 0 | 0 | 0 | 2 |
| Glo20 | 0 | 0 | 0 |  | 0 | 0 | 2 |  | 0 | 0 | 0 | 2 |
| Glo21 | 0 | 0 | 0 |  | 0 | 0 | 2 |  | 0 | 0 | 0 | 2 |
| Glo22 | 62 | 11 | 11 |  | 24 | 9 | 3 |  | 17 | 1 | 46 | 184 |
| Aca1 | 2 | 0 | 0 |  | 0 | 0 | 0 |  | 0 | 0 | 0 | 2 |
| **Total** | 84 | 82 | 85 |  | 82 | 87 | 87 |  | 82 | 84 | 88 | 761 |

Note: Ai, Hl and Aa represent the *Acanthus ilicifolius*, *Heritiera littoraliss* and *Acrostichum aureum*, respectively; HTL, MTL and LTL represent the high, middle and low tide level, respectively.
