# Supplementary material for: Flooding Greatly Affects the Diversity of Arbuscular Mycorrhizal Fungi Communities in the Roots of Wetland Plants
Source: PLoS One. 2011 Sep 12;6(9):e24512. doi: 10.1371/journal.pone.0024512 (PMC3171463; doi:10.1371/journal.pone.0024512)
Supplement: Table S2 — BLAST results for the unidentified environmental sequences from GenBank related to the phylotypes obtained in this study. (DOC) [file pone.0024512.s004.doc]

**Table S2 BLAST results for the unidentified environmental sequences from GenBank related to the phylotypes obtained in this study. The ITS region of Glo5, Glo7, Glo9, Glo13 and Glo18, which should come from different AMF species (see Figure 2), were all related to the same environmental sequences, implying that it is not possible to use the ITS region alone for species-level discrimination of all AMF.**

| Phylotypes |  | GenBank code |  | Fragment |  | Similarity |  | Host plant and geographic origin |
| --- | --- | --- | --- | --- | --- | --- | --- | --- |
| Glo5 |  | FM206335 |  | ITS |  | 96% |  | *Dioscorea rotundata* root, Guinea |
| Glo7 |  | FM206335 |  | ITS |  | 95% |  | ─ |
| Glo9 |  | FM206335 |  | ITS |  | 94% |  | ─ |
| Glo13 |  | FM206335 |  | ITS |  | 95% |  | ─ |
| Glo18 |  | FM206335 |  | ITS |  | 96% |  | ─ |
| Glo21 |  | AB369765 |  | LSU |  | 97% |  | *Miscanthus sinensis* root, Japan |
